# GDNF and the RET Receptor in Cancer: New Insights and Therapeutic Potential

**DOI:** 10.3389/fphys.2018.01873

**Published:** 2019-01-07

**Authors:** Lois M. Mulligan

**Affiliations:** Division of Cancer Biology and Genetics, Department of Pathology and Molecular Medicine, Cancer Research Institute, Queen’s University, Kingston, ON, Canada

**Keywords:** GDNF, RET, oncogene, tumor microenvironment, targeted therapies

## Abstract

The Glial cell line-derived neurotrophic Family Ligands (GFL) are soluble neurotrophic factors that are required for development of multiple human tissues, but which are also important contributors to human cancers. GFL signaling occurs through the transmembrane RET receptor tyrosine kinase, a well-characterized oncogene. GFL-independent RET activation, through rearrangement or point mutations occurs in thyroid and lung cancers. However, GFL-mediated activation of wildtype RET is an increasingly recognized mechanism promoting tumor growth and dissemination of a much broader group of cancers. RET and GFL expression have been implicated in metastasis or invasion in diverse human cancers including breast, pancreatic, and prostate tumors, where they are linked to poorer patient prognosis. In addition to directly inducing tumor growth in these diseases, GFL-RET signaling promotes changes in the tumor microenvironment that alter the surrounding stroma and cellular composition to enhance tumor invasion and metastasis. As such, GFL RET signaling is an important target for novel therapeutic approaches to limit tumor growth and spread and improve disease outcomes.

## Introduction

The neurotrophins are a family of soluble neurotrophic factors, originally recognized for their abilities to regulate growth, survival and differentiation of neural-derived cell types. Neurotrophins have well characterized roles as guidance, survival and differentiation factors in developing neurons in the central (CNS) and peripheral nervous systems (PNS) and may also promote survival or regrowth of mature neurons, by binding their cell-surface receptors and stimulating downstream signals in their target cells. However, with the increasing availability of tissue and cell-specific transcriptome and proteome data, it is becoming clear that neurotrophic factors and their receptors are also broadly expressed on other, non-neural cell types, where they can contribute to cell growth, differentiation and migration and tissue maturation. Importantly, the aberrant expression or activation of these signaling complexes can allow these normal growth signals to contribute to the growth or spread of cancer cells, making expression and functions of neurotrophins and their receptors important contributors to human cancer, and potentially valuable therapeutic targets.

## The GDNF Family and RET Receptor

**FIGURE 1:**
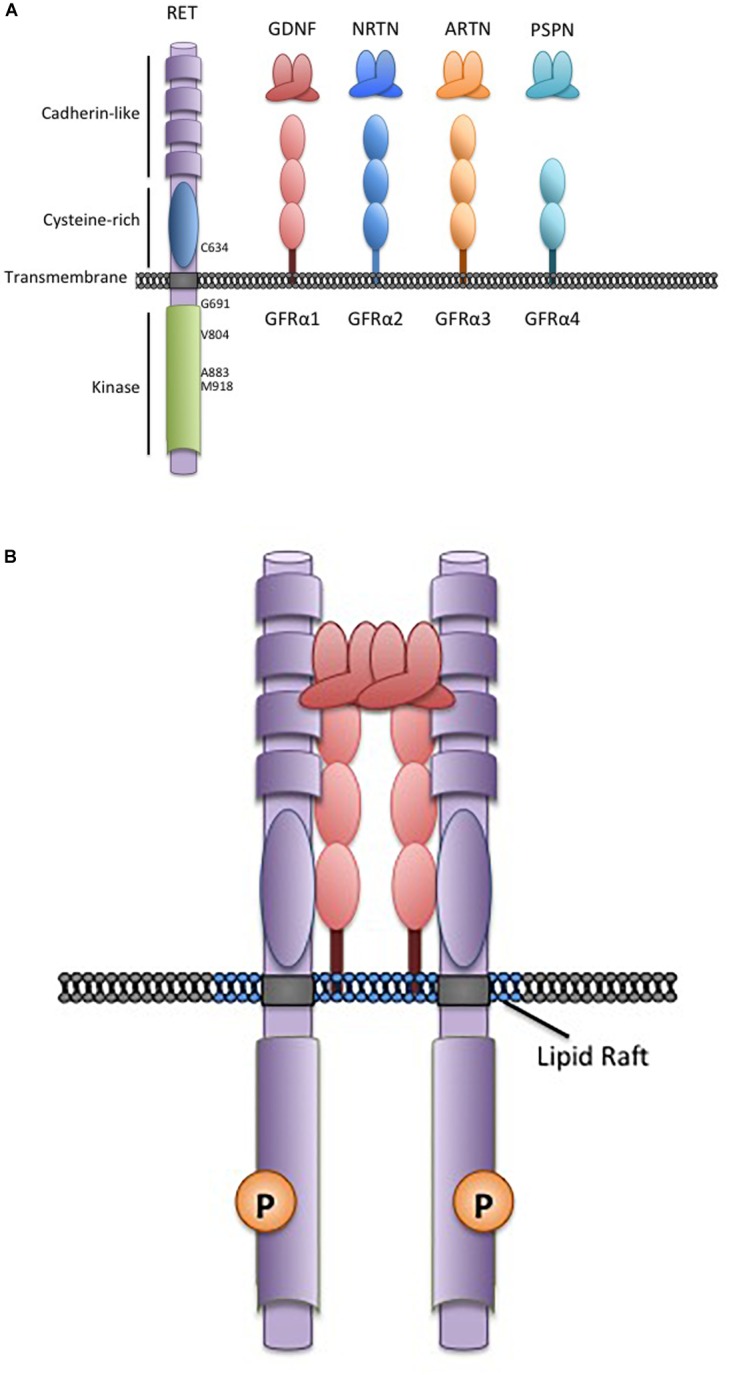
The RET receptor, associates with GDNF family ligands and coreceptors. **(A)** The RET receptor tyrosine kinase showing its major functional domains. RET is the transmembrane receptor for the four GFLs. GFLs are recruited to RET by interactions with cell surface coreceptors of the GFRα family, consisting of either two or three globular protein domains tethered to the cell membrane by a GPI-linkage. Locations of key RET residues are indicated. **(B)** GFL-RET signaling complexes. GFL and GFRα family members associate in a 2:2 ratio (Parkash et al., 2008). Theses complexes recruit RET to lipid raft membrane domains where signaling occurs (Tansey et al., 2000).

The Glial Cell-line Derived Neurotrophic Factors (GDNF) are a family of neurotrophins with similarities to the transforming growth factor β growth regulatory proteins. There are four structurally similar family members: GDNF, neurturin (NRTN), artemin (ARTN), and persephin (PSPN) that are recruited to corresponding non-signaling co-receptors of the GDNF Family Receptors α (GFRα1-4), which are tethered to the plasma membrane through glycosylphosphatidylinositol-anchors (Airaksinen and Saarma, 2002; Figure 1). GDNF Family Ligands (GFL) and GFRα family members have distinct but overlapping tissue-specific expression patterns that determine their biological roles, however, all GFL-GFRα complexes signal through a single transmembrane receptor, the RET (REarranged during Transfection) tyrosine kinase (Mulligan, 2014). GFL-GFRα complexes associate with RET’s large extracellular domain, promoting dimerization and activation of its intracellular kinase domain, leading to stimulation of multiple downstream pathways (Mulligan, 2014; Figure 1). In early development, GFL-RET signals promote proliferation and migration of neural crest-derived cells to populate neuroendocrine organs and contribute to the development of central and peripheral nerve lineages, most notably the enteric nervous system (Avantaggiato et al., 1994; Schuchardt et al., 1994). In the genitourinary system, activation of RET receptor signaling is essential for growth and formation of the kidney, and later for maturation of spermatogonia (Schuchardt et al., 1996; Meng et al., 2000). More recently, important roles for GFL-RET in formation of the Peyer’s patches, lymphoid structures in the gut, and the maintenance and expansion of hematopoietic stem cells have been recognized (Vargas-Leal et al., 2005; Veiga-Fernandes et al., 2007; Fonseca-Pereira et al., 2014). However, the developmentally important processes of cell proliferation and migration and stem cell renewal can also be “hijacked” to promote the growth and spread of cancer. RET is a well characterized contributor to the neoplastic process, acting as an oncogenic driver in several cancers, and has been more recently recognized as a critical determinant of invasion and spread in diverse tumor types (Mulligan, 2014). These processes can be GFL-independent or -dependent, with variations depending on the type of tumor and the mechanisms leading to stimulation of RET receptor signaling. Although GFL-GFRα complexes have been demonstrated to induce intracellular signaling through other receptors (Paratcha et al., 2003; Popsueva et al., 2003), their role in RET-associated processes is best described in the cancer setting and will be the focus here.

## RET Dependent, GFL Independent Cancers

Oncogenic mutations that constitutively activate the RET receptor in the absence of GFLs have been recognized for over 30 years (Fusco et al., 1987; Mulligan et al., 1993). Clinically, screening for these mutations can be a valuable tool in establishing differential diagnosis and guiding disease management. RET mutations fall into two distinct classes based on mutational mechanisms and are consistently associated with distinct tumor types.

### *RET* Gene Rearrangements

Somatic rearrangements of the *RET* gene, resulting from chromosomal rearrangements or inversions, lead to the juxtaposition of the RET intracellular kinase domain sequences with N-terminal sequences from another protein which contain dimerization domains such as coiled-coil motifs (Romei et al., 2016). RET fusions have been found in 10–20% of papillary thyroid carcinoma (PTC), ∼1–2% of non-small-cell lung carcinoma (NSCLC), and more recently, in 3% of Spitzoid tumors (Wiesner et al., 2014; Romei et al., 2016; Drilon et al., 2018a; Liang et al., 2018). Increasingly, deep sequencing approaches on a wider variety of tumors are identifying less frequent *RET* rearrangements in other cancer types including chronic myelomonocytic leukemia and colorectal, breast, ovarian and head and neck tumors (Ballerini et al., 2012; Kato et al., 2017; Gozgit et al., 2018; Mulligan, 2018; Paratala et al., 2018; Pietrantonio et al., 2018; Skalova et al., 2018).

The chimeric RET fusion proteins generated in response to these rearrangements, localize in the cytosol and are constitutively active in the absence of any GFL. Because of their location, they are able to escape many of the processes that regulate a membrane-associated RET protein and promote sustained activation of downstream survival and growth pathways (Richardson et al., 2009; Xing, 2013). Over 30 RET fusion partner genes have been identified to date, and the distribution of different partners varies amongst tumor types. For example, the *kinesin family 5B-RET* gene rearrangement is the most commonly found in lung adenocarcinoma but is rare in other tumor types, while *coiled-coil domain containing 6-RET* rearrangements are common in PTC and several tumor types (Romei et al., 2016; Gautschi et al., 2017; Ferrara et al., 2018).

Identification of *RET* mutations can provide valuable prognostic and predictive tools to guide patient management. In PTC, *RET* rearrangements appear to be an early event in tumourigenesis (Viglietto et al., 1995). Although not essential for PTC formation, presence of a RET fusion protein or increased expression is linked to more malignant phenotypes including regional invasion and lymph node metastasis (Miki et al., 1999; Wang et al., 2008; Yip et al., 2015; Khan et al., 2018). Thus, RET rearrangements are an important marker for risk of malignancy in fine needle aspirates of thyroid nodules with indeterminate cytology. As part of a positive predictive multigene panel (e.g., ThyroSec v3, or ThyGenX/ThyraMIR), recognition of *RET* variants can help to distinguish benign lesions from cancer and identify cases requiring surgery (Sapio et al., 2007; Onenerk et al., 2017; Paschke et al., 2017; Steward et al., 2018). In contrast to PTC, *RET* mutations appear to be oncogenic drivers in NSCLC, and are more common in younger never-smokers (Wang R. et al., 2012; Tsuta et al., 2014; Gautschi et al., 2017). Although RET rearrangements are infrequent, screening as part of a multigene panel or in patients where other lung cancer genes have been excluded, is recommended to identify patients who may benefit from RET targeted therapies (below) (Kalemkerian et al., 2018). Interestingly, recent studies indicate that increased expression of wildtype RET occurs in an even larger pool of NSCLC, where it may be linked to poor tumor differentiation (Tan et al., 2018), suggesting that, in addition to RET fusions, GFL-RET signaling may also contribute to these tumors.

### *RET* Point Mutations

In contrast, gain-of-function point mutations in the RET receptor give rise to multiple endocrine neoplasia 2 (MEN2), an inherited cancer syndrome characterized by medullary thyroid cancers (MTC) and the adrenal tumor pheochromocytoma (Mulligan, 2014; Wells, 2018). Similar mutations also occur somatically in 40–65% sporadic MTC where they are an important biomarker that identifies more aggressive disease (Mulligan, 2014; Vuong et al., 2018; Wells, 2018). Interestingly, unlike *RET* rearrangements, activating *RET* point mutations are extremely rare outside of the neuroendocrine tumors. In MEN2, mutations in the RET extracellular domain result in constitutive dimerization and activation, while mutations of intracellular sequences generally affect kinase autoinhibition or ATP-binding and facilitate activation of RET monomers (Mulligan, 2014; Plaza-Menacho, 2018). As a result, full-length MEN2-RET receptors at the cell membrane are constitutively active in the absence of GFLs and stimulate unregulated signaling through pathways associated with wildtype RET activity (Asai et al., 1995; Santoro et al., 1995). However, because these active RET forms localize on the cell surface, they can also associate with GFL-GFRα complexes, which may further enhance their oncogenic activity (Bongarzone et al., 1998; Cranston et al., 2006; Gujral et al., 2006). There are strong associations of MEN2 disease phenotype with specific *RET* mutations, with more severe disease associated with relatively greater increases in mutant RET kinase activity (Eng et al., 1996; Wells et al., 2015). These associations provide the basis of clinical management guidelines for patients with MEN 2. Genetic screening for *RET* mutations as early as possible is now the standard of care for all at-risk individuals, allowing recommendations to be tailored to the risks associated with specific *RET* mutations (Wells et al., 2015). For example, a specific methionine to threonine change at codon 918 (M918T) in the RET kinase domain (Figure 1), leads to a 10-fold increase in ATP-binding and RET kinase activity and is associated with the earliest disease onset and poorest prognosis (Gujral et al., 2006; Wells et al., 2015). In these cases, thyroidectomy is recommended in the first year of life, to minimize risks of MTC micrometastasis (Sanso et al., 2002; Zenaty et al., 2009; Wells et al., 2015). In patients with other “high risk” RET mutations (e.g., C634R, A883F) surgery may be delayed within the first 5 years, while a combination of biochemical monitoring and delayed surgery may be appropriate for families with other more moderate mutations and later familial disease onset (Wells et al., 2015; Voss et al., 2017). The effects of RET mutations in familial and sporadic cancers and further details of specific genotype-phenotype correlations have been well documented in recent reviews (Romei et al., 2016; Drilon et al., 2018a; Mulligan, 2018).

## GFL-Mediated RET Activity in Cancer

In addition to oncogenic mutations, increased expression or activity of wildtype RET is now being recognized in many additional tumor types where it may have a range of different implications. In response to GFLs, which are released at some level by many tumors as well as by many cell types in the tumor microenvironment, RET signaling can enhance growth, promote tumor spread or even affect response to therapies.

### Breast Cancer

RET and GFRα1 are expressed in approximately 30–70% of human breast cancers (Esseghir et al., 2007; Boulay et al., 2008; Morandi et al., 2011). Recent data have suggested that RET variants or amplification may also occur in ∼1% of breast tumors or metastases (Paratala et al., 2018), while tumor-specific expression of GDNF and ARTN is relatively frequent and can promote autocrine activation of RET downstream signaling (Kang et al., 2009; Kan et al., 2010; Morandi et al., 2013; Nik-Zainal et al., 2016). RET expression is more common in estrogen receptor positive (ER+ve) tumors, which are the most common breast cancer subtype, but it is also detected in other subtypes (Morandi et al., 2011; Gattelli et al., 2013). Expression is higher in recurrent cancers compared to normal tissues and corresponding primary tumors and is correlated with larger tumor size, higher tumor stage and reduced metastasis-free and overall survival (Esseghir et al., 2007; Boulay et al., 2008; Plaza-Menacho et al., 2010; Wang C. et al., 2012; Gattelli et al., 2013; Morandi et al., 2013). In *in vitro* cell and animal models of breast cancer, activation of RET signaling by GFLs enhances tumor cell proliferation, and survival and has been shown to promote estrogen-independent expression of a normally estrogen-ER-dependent transcriptional profile (Esseghir et al., 2007; Boulay et al., 2008; Plaza-Menacho et al., 2010; Wang C. et al., 2012; Gattelli et al., 2013, 2018). Both the *RET* and *GFRA1* genes are also positively regulated by estrogen-ER, suggesting a potential feedback loop enhancing growth in ER+ve tumors (Stine et al., 2011; Horibata et al., 2018).

Importantly, RET expression in breast cancer is also correlated with resistance to endocrine therapies via stimulation of the mTOR signaling pathway (Plaza-Menacho et al., 2010; Gattelli et al., 2013; Morandi et al., 2013). Treatments that target tumor responses to estrogen, including selective estrogen receptor modulators (e.g., tamoxifen), agents blocking estrogen biosynthesis (e.g., aromatase inhibitors), and estrogen receptor antagonists (e.g., fulvestrant) are an important adjuvant to first line surgery, chemotherapy or radiation for women with ER+ve breast cancers (Pagani et al., 2010; Liu et al., 2017). Acquired or intrinsic endocrine resistance to these therapies limits options to block tumor growth or recurrence in these patients (Liu et al., 2017). RET expression is frequently elevated in ER+ve tumors, and patients with RET +ve tumors are over represented amongst cases with acquired endocrine resistance, suggesting a functional link (Plaza-Menacho et al., 2010; Morandi et al., 2013). Recent data suggest that upregulation of GFL ligands in these tumors may facilitate the transition of these “predisposed” RET-expressing tumors to endocrine resistance (Horibata et al., 2018). Excitingly, several recent studies in preclinical animal models have suggested that targeting RET function could both limit tumor growth and resensitize tumors to endocrine therapies, prolonging the efficacy of these agents (Nguyen et al., 2015; Andreucci et al., 2016; Hatem et al., 2016; Gattelli et al., 2018).

### Pancreatic Cancer

RET and GFRαs are expressed in 40–65% of pancreatic ductal adenocarcinomas (PDAC) and are an indicator of poorer prognosis and reduced overall survival (Zeng et al., 2008; He et al., 2014). Expression is more frequent in high grade and metastatic tumors and in patients with lymphatic and perineural invasion (Ito et al., 2005; Zeng et al., 2008). The pancreatic environment is rich in GDNF and ARTN, which are robustly secreted by PDACs themselves but also by intra- and extra-pancreatic nerves, and by macrophages in the perineural space (Ito et al., 2005; Zeng et al., 2008; Gil et al., 2010; Amit et al., 2017). Secretion of GFLs and soluble forms of GFRα1 within the nerve, stimulates movement of RET-expressing tumor cells along a chemotactic gradient of GFLs to invade the perineural space and move along nerve fibers toward the central nervous system, an invasive process which is strongly linked to poorer patient outcomes, neuropathy and tumor-related pain (Veit et al., 2004; Zeng et al., 2008; Gil et al., 2010; Cavel et al., 2012; He et al., 2014; Amit et al., 2016).

Interestingly, although activating RET mutations are very rare in PDAC, a common polymorphic glycine to serine sequence variant at codon 691 (G691S) in the RET intracellular juxtamembrane region is over represented in PDAC patients (∼20% cases) and has been linked to increased GDNF-dependent proliferation and invasion (Sawai et al., 2005), suggesting this variant may act as a risk allele to modify RET function.

### Prostate Cancer

RET protein has been detected in 20–75% of high grade prostate adenocarcinomas (Gleason score >3) while GDNF is upregulated in both prostate tumors and surrounding stroma (Dawson et al., 1998; Dakhova et al., 2009; Ban et al., 2017). GDNF-mediated RET activity also promotes cell proliferation, invasion and perineural spread in *in vitro* and preclinical animal models of prostate cancer and can be further enhanced in the presence of soluble forms of GFRα1 released by nerves (Gil et al., 2010; Liu et al., 2012; Ban et al., 2017). Upregulation of RET and GDNF is associated with increased expression of matrix degrading metalloproteinases that facilitate the invasion of tumor cells into the perineural space (Baspinar et al., 2017). Importantly, GDNF is robustly secreted by fibroblasts in the tumor environment upon treatment-induced DNA damage, which can promote prostate cancer cell proliferation and may act as a feedback mechanism contributing to treatment resistance (Huber et al., 2015).

### Colorectal Cancer

The effects of GFL-mediated RET activation in colorectal cancer are less clear. RET expression is relatively low in colon adenocarcinoma and several studies have noted frequent methylation of the RET promoter, suggesting reduced RET expression may be associated with worse prognosis in some cases (Luo et al., 2013; Draht et al., 2014). Interestingly, ARTN, NRTN, GFRα1, and GFRα3 are found in colon tumors, potentially arising from gut nerves or associated with chronic inflammation of the intestine (Qiao et al., 2009; Luo et al., 2013; Han et al., 2015), which increases cancer risk, suggesting a subset of RET-expressing colon tumors may be responsive to GFL stimulation (Mendes Oliveira et al., 2018). Increasingly, whole genome profiling or focused mutation panels are identifying *RET* rearrangements in colorectal cancers similar to those in thyroid and lung (Le Rolle et al., 2015; Kloosterman et al., 2017; Mendes Oliveira et al., 2018; Pietrantonio et al., 2018). RET chimeric oncoproteins are rare (0.2–0.5% cases), but promote tumor growth and migration in animal and cell based models (Gozgit et al., 2018) and have been correlated with worse prognosis, poor treatment response and reduced overall survival in colon adenocarcinoma patients (Le Rolle et al., 2015). Together, these data suggest that at least in some cases, RET may act as an oncogenic driver in colon as well. Thus, further investigation is required to establish significance of RET and GFL activity in colorectal cancer.

### Myeloid Malignancies

RET is expressed in myeloid tumors but is rare in lymphoid tumors (Gattei et al., 1997). Increased RET activity, mediated through NRTN or ARTN ligand complexes secreted by stromal cells, is detected in 60–70% of Acute Myeloid Leukemia (AML) cases with myelomonocytic differentiation, where it may promote cell viability and proliferation through suppression of autophagy by mTORC1-mediated signals (Gattei et al., 1997, 1998; Camos et al., 2006; Rudat et al., 2018). Expression is higher in AML cases with worse prognosis (Yu et al., 2015). In patients with AML associated with a t(8;16)(p11;p13) translocation, increased RET expression may be a result of altered levels of a group of miRNAs predicted to regulate its normal expression (Diaz-Beya et al., 2013). In a single study, rare RET rearrangements have been found in patients with the myeloproliferative disorder, chronic myelomonocytic leukemia (Ballerini et al., 2012).

### Other Cancers

Elevated levels of GFL and RET expression, without mutation, are detected in a subset of several other cancers including: melanoma, glioma, neuroblastoma, seminoma, endometrial, and head and neck cancers and renal cell carcinomas (Wiesenhofer et al., 2000; Narita et al., 2009; Pandey et al., 2010; Flavin et al., 2012; Chuang et al., 2013; Kosari et al., 2014; Lin et al., 2016). In many of these diseases, RET appears to stimulate tumor cell migration or invasion and is correlated with reduced overall survival (Narita et al., 2009; Pandey et al., 2010; Chuang et al., 2013; Kosari et al., 2014; Lin et al., 2016; Pietrantonio et al., 2018). Intriguingly, RET and GFL expression is found even more broadly in cancer cell lines from various tissues, although *in vivo* correlates of this in primary tumors are not always available and significance in some cases is not yet clear (Fielder et al., 2018). Together, these data suggest that targeting RET may be therapeutically valuable in a broader and more diverse group of human cancers than recognized to date.

## GFL-RET Modulation of the Tumor Microenvironment

**FIGURE 2:**
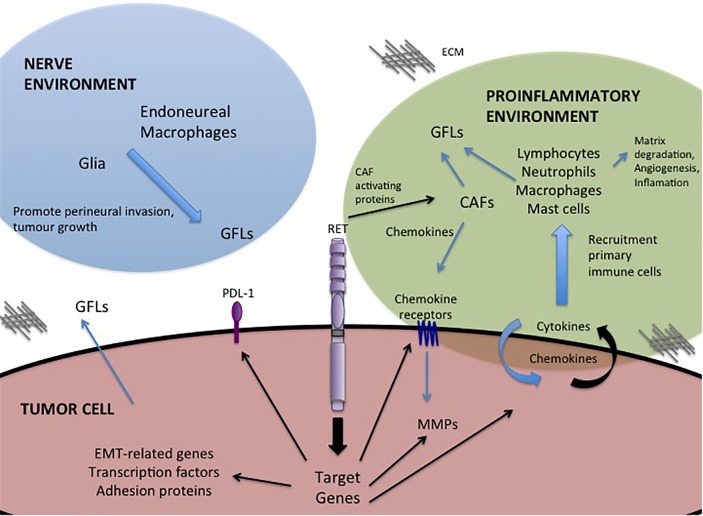
GFL-mediated influences in the tumor microenvironment. Activation of RET receptor signaling in tumor cells promotes expression of a portfolio of proteins that regulate interactions with the tumor microenvironment. RET activity promotes changes in the composition of the tumor environment and stimulates autocrine and paracrine signals to recruit immune cells, remodel the extracellular matrix (ECM) and promote invasion toward the neural environment. Black arrows – Direct targets of RET activity.

One of the most important roles of GFL-RET signaling in cancer is in modulating the relationship between the tumor and its surroundings. The tumor microenvironment is complex, comprising vessels (blood and lymph), and a plethora of cellular components including cancer-associated fibroblasts (CAF), stromal, and immune or inflammatory cells all surrounded and supported by non-cellular components of the extracellular matrix (ECM) (Figure 2). Interactions of tumor cells and their environment can result in reciprocal remodeling that enhances the ability of the tumor to grow, and invade surrounding tissues or escape immune response and initiate metastasis. In multiple cancers, stimulation of RET activity leads to changes in expression of transcription factors (e.g., SLUG, SNAIL, ZEB, TWIST), adhesion proteins (e.g., E-cadherin, N-cadherin, vimentin) and matrix remodeling proteins (e.g., matrix metalloproteases) that can cause cancer cells to take on more mesenchymal phenotypes (Melillo et al., 2005; Lian et al., 2017; Castellone and Melillo, 2018). Cells undergoing RET-mediated epithelial to mesenchymal transition (EMT) remodel their actin cytoskeleton and lose cell polarity, becoming more motile, and have enhanced abilities to degrade the ECM to promote cell invasion (Tang et al., 1998; Asai et al., 1999; Melillo et al., 2005; Lian et al., 2017).

Importantly, RET is implicated in promoting tumor-related inflammation, the infiltration of immune cells into the tumor environment, a key indicator of disease outcomes and therapeutic responses (Borrello et al., 2008). RET activation, either by oncogenic mutations or GFL stimulation, has been shown to contribute to this process by inducing expression of proinflammatory proteins including cytokines, chemokines and their receptors (Borrello et al., 2005; Puxeddu et al., 2005; Cavel et al., 2012; Menicali et al., 2012; Gattelli et al., 2013; Rusmini et al., 2014; Figure 2). When released, these molecules may act directly on the tumor cell, leading to an autocrine loop that further enhances tumor growth or motility (e.g., CXCR8/IL8), or they may promote changes in the tumor microenvironment, acting as chemoattractants for primary immune cells (lymphocytes, neutrophils, macrophages, mast cells) (Figure 2) that infiltrate the growing tumor, which in turn contribute to matrix degradation, angiogenesis and increased inflammation (Borrello et al., 2005; Cavel et al., 2012; Gattelli et al., 2013). For example, RET activity in thyroid, breast and pancreatic cancers can enhance tumor expression of chemokine receptor CXCR4, triggering responses to circulating chemokines released by CAFs or the tumor itself, to recruit endothelial progenitors that promote angiogenesis to sustain the growing tumor mass (Castellone et al., 2004; Borrello et al., 2005; Lu et al., 2011; Werner et al., 2017; Wang et al., 2018). Inflammatory cytokines can also promote release of GFLs by infiltrating macrophages or fibroblasts (Figure 2), particularly in the perineural environment, to further enhance RET-mediated effects (Vargas-Leal et al., 2005; Esseghir et al., 2007; Cavel et al., 2012). Several studies have shown that varying levels of RET are expressed broadly on immune cells of both myeloid and lymphoid lineages, suggesting that recruitment of these cells to the tumor microenvironment may be enhanced by higher localized GFL levels, further potentiating tumor growth and invasive spread (Borrello et al., 2005; Vargas-Leal et al., 2005; Cavel et al., 2012; Gattelli et al., 2013; Rusmini et al., 2014; Ibiza et al., 2016). Together, these data reveal a complex web of autocrine and paracrine stimulatory signals that promote remodeling of the tumor environment, facilitating the oncogenic potential of RET-expressing tumors.

## GFL-RET and the Therapeutic Landscape

As recognition of RET’s impact in diverse cancers expands, it has become an increasingly important therapeutic target. As yet, there are no agents that specifically target the RET kinase in clinical use, however, multikinase inhibitors originally developed against other kinases that also inhibit RET activity are proving valuable (Drilon et al., 2018a; Redaelli et al., 2018). Specifically, two tyrosine kinase inhibitors (TKI), the VEGFR2/EGFR inhibitor vandetanib and VEGFR2/MET inhibitor cabozantinib, are approved for treatment of advanced thyroid cancer, and have been evaluated in clinical trials for RET-associated lung adenocarcinoma (Wells et al., 2012; Drilon et al., 2016, 2018a; Schlumberger et al., 2017; Yoh et al., 2017). Treatment with these TKIs has yielded significant improvements in progression free survival in MTC patients with activating RET mutations, leading to stable disease or extended response duration (Wells et al., 2012; Schlumberger et al., 2017). Notably, improved overall survival has been reported for patients with MTC harboring the RET M918T mutation, characteristic of the most aggressive form of MEN2, but is not significantly increased for patients with other RET mutations (Fox et al., 2013; Schlumberger et al., 2017). In NSCLC with RET mutations, partial responses have been reported but clinical benefit has been limited and these agents have not to date significantly improved patient outcomes (Drilon et al., 2016; Gautschi et al., 2017; Yoh et al., 2017; Ferrara et al., 2018). Similarly, in breast cancer, RET-targeting TKIs have not shown significant benefit, although previous studies have not specifically focused on RET-positive tumors (Miller et al., 2005; Bronte et al., 2017).

Further, these approved multikinase inhibitors are associated with an array of significant off-target side effects, likely due to inhibition of other kinase family members (Drilon et al., 2018a). A number of other multikinase TKIs (e.g., ponatinib, alectinib, sorafenib, lenvatinib, RXDX-105) are currently in clinical trials or early preclinical testing for RET-associated cancers (Drilon et al., 2018a; Redaelli et al., 2018). Like vandetanib and cabozantinib, these are primarily ATP-competitive inhibitors that bind conserved residues at the ATP-binding site of the kinase (Roskoski and Sadeghi-Nejad, 2018). Interestingly some of the amino acid substitution RET mutations found in MEN2 involve these “gate-keeper” residues (e.g., V804M), which determine inhibitor “fit” within the ATP-binding pocket, altering the ability of some TKIs to bind and inhibit RET (e.g., vandetanib, motesanib) (Carlomagno et al., 2004; Redaelli et al., 2018). Each of the inhibitors currently being explored has distinct abilities to inhibit RET kinase domain mutants (Liu et al., 2018). Thus, not all TKIs perform equally well at inhibiting RET mutants and patient genotype is an important determinant of optimal therapy (Carlomagno et al., 2004; Redaelli et al., 2018). Further, recent reports of acquired resistance, due to somatic mutations of this same residue, in patients treated with TKIs (Subbiah et al., 2018c), suggest that the development of novel, more selective RET inhibitors has many advantages.

A new generation of kinase inhibitors with improved selectivity for RET that also efficiently inhibit the activity of wildtype and all of the known RET mutants, are now coming to the fore. A number of promising RET-selective agents are currently in preclinical evaluation and early clinical trials (Drilon et al., 2018a; Redaelli et al., 2018). Two of the most exciting of these, BLU-667 and LOXO-292, have more than 100-fold greater selectivity for RET compared to other kinases (Subbiah et al., 2018b,c) and are currently being evaluated in clinical trials for RET-associated lung, thyroid, colon and other solid tumors^1^. Early reports suggest these agents are better tolerated, with fewer off-target effects than multikinase inhibitors (Drilon et al., 2018c; Subbiah et al., 2018b,c). Preliminary results of a Phase I trial of LOXO-292, report high response rates for patients with RET fusion proteins (∼69%) and also suggest some effect on brain metastases, a key challenge in managing NSCLC (Drilon et al., 2018b,c; Subbiah et al., 2018c). These studies have led to FDA designation of LOXO-292 as a breakthrough therapy for RET-mutation positive thyroid carcinoma and NSCLC.

While RET-selective agents are improving targeting of RET-associated cancers now, in the future, additional approaches and combinations of therapies will further expand options. Combination therapies, coupling RET inhibitors with other therapeutic approaches may further enhance patient outcomes. Several studies, combining inhibitors of RET and mTOR signaling (e.g., everolimus) have shown increased or prolonged benefit over single agents in thyroid and breast cancer models (Plaza-Menacho et al., 2010; Gild et al., 2013; Subbiah et al., 2015). Excitingly, early reports from a clinical trial in lung cancer suggests that this combination may also improve delivery of RET inhibitor across the blood-brain barrier, essential for treating brain metastases (Plaza-Menacho et al., 2010; Gild et al., 2013; Subbiah et al., 2015, 2018a).

Alternative strategies to target the GFL-RET axis are also under development. Antibody-drug conjugates targeted to RET or GFRα1, have demonstrated effective and specific killing of breast cancer cells *in vitro* and *in vivo* (Nguyen et al., 2015; Bhakta et al., 2017; Bosco et al., 2018). Recent studies have capitalized on adoptive T-cell immunotherapy approaches to develop GFRα4-targeting chimeric antigen receptor (CAR)-modified T cells that can promote cytotoxicity and limit growth of MTC cell lines in animal models (Bhoj et al., 2016). Interestingly, GDNF also upregulates the immune inhibitory factor Programmed Death Ligand (PDL-1) in some tumors (Lin et al., 2017; Figure 2), which when bound to its PD-1 receptor can mediate suppression of local immune responses, leading to immune evasion by tumor cells (Alsaab et al., 2017). Recent advances in cancer immunotherapy targeting the PDL-1/PD-1 immune checkpoint to release the blockade of immune responses, have shown dramatic promise as an adjuvant to established chemo or radio therapies in patients with deficiencies of DNA repair and high mutational burden (Gong et al., 2018). However, preliminary data in lung have shown low mutational burden and minimal response to immunotherapies in tumors bearing *RET* rearrangements, suggesting that this approach alone may not have significant benefits in this subgroup of tumors and should be coupled to other standard or targeted therapies (Sarfaty et al., 2017; Sabari et al., 2018).

## Non-Tumor Effects of GFL-RET Targeted Therapies

Despite the promise of personalized therapy for RET-associated cancers, the longer term effects of RET inhibition in mature normal tissues will need to be carefully considered. GFL-RET signaling is essential in development of nervous and hematopoietic systems, but also has important roles in the maintenance and survival of mature nerve lineages in the CNS and PNS. Prolonged inhibition of these signals may compromise nerve health and survival, particularly in aged neurons or in response to stress or damage (Kramer et al., 2007; Meka et al., 2015; Soba et al., 2015). Although many TKIs have limited abilities to cross the blood-brain barrier, more recent TKI and RET selective inhibitors (e.g., LOXO-292) have improved penetration into the CNS, where GFL-RET signals are important survival factors for dopaminergic neurons (Lin et al., 1993; Drinkut et al., 2016). Thus, until the long-term effects of blocking CNS GFL-signaling in the cancer setting are clear, optimal treatment of intracranial metastasis and maintaining nerve health may need to be carefully balanced.

GFL-RET signals are also important for hematopoietic stem cell maintenance and expansion in adult animals, suggesting that reduced levels as a result of treatment could impact the portfolio of immune cells generated and responses to immune challenge (Fonseca-Pereira et al., 2014). In animal models, RET ablation impairs gut homeostasis and increases the risks of inflammation or infection in the gut in response to irritants (Ibiza et al., 2016), which could also impact patient ability to remain on RET inhibitor treatment.

Finally, GFL-RET signaling has also been implicated in tumor related pain, but this relationship appears complex. GFL and RET expression are correlated with perineural invasion and resultant increased pain levels in pancreatic cancer patients (Wang et al., 2014; Amit et al., 2016). However, reports are quite variable on whether GFLs increase or decrease cancer or inflammation-related bone pain (Golden et al., 2010; Ding et al., 2017; Nencini et al., 2018). It is likely that variations in the underlying mechanisms causing pain, and potentially central versus peripheral responses, may contribute to these differences. Likewise, the effect of GFL-RET-targeted therapies on tumor pain may vary with cancer type and degree or sites of dissemination.

## Future Considerations for GFL-RET in Cancer

While RET receptor mutations are well-characterized mechanisms of carcinogenesis, the much broader implications of GFL-mediated RET signaling in cancer are only beginning to be recognized. State-of-the-art diagnostic approaches, such as liquid biopsies coupled to Next Generation Sequencing, or high through-put mutational screening panels to assess circulating tumor cells or cell free tumor DNA (Hench et al., 2018; Vaughn et al., 2018), are increasing our abilities to recognize RET-targetable cancers or recurrent disease, without need for more invasive tumor biopsies (Reckamp et al., 2018).

With more intensive scrutiny of cancer genomes, *RET* mutations continue to be recognized, but many tumor types are now also being found to respond to GFLs released in the microenvironment by the tumor itself or neighboring cells, expanding the range of pathologies that may benefit from targeting this pathway. GFL-RET signaling both promotes inflammation in the tumor microenvironment and enhances tumor responses to it, which may provide an additional mechanism increasing proliferation and dissemination of diverse cancers. Interestingly, recent studies have also linked RET to alterations in tumor metabolism, an emerging hallmark of cancer, through a novel ligand complex involving Growth Differentiation Factor 15 (GDF15) and the GDNF Family Receptor α-like (GFRAL) (Emmerson et al., 2017; Hsu et al., 2017; Mullican et al., 2017; Yang et al., 2017). GDF15-GFRAL-RET regulates metabolic homeostasis, particularly under stress conditions, but may also promote cancer associated anorexia or cachexia (Johnen et al., 2007; Lerner et al., 2015), suggesting that blocking RET signals may have added benefit in reducing weight loss associated with other forms of therapy. Together, these data highlight the complexity of GFL-RET signaling and the potential benefits and challenges of new therapeutic strategies for targeting this pathway that are rapidly transitioning to the clinic, to change cancer management and improve patient outcomes.

## Author Contributions

LM prepared the manuscript.

## Conflict of Interest Statement

The author declares that the research was conducted in the absence of any commercial or financial relationships that could be construed as a potential conflict of interest.

## Footnotes

1 clinicaltrials.gov
